# Antibodies to PfEMP1 and variant surface antigens: Protection after controlled human malaria infection in semi-immune Kenyan adults

**DOI:** 10.1016/j.jinf.2024.106252

**Published:** 2024-10

**Authors:** Ann W. Kinyua, Louise Turner, Hannah W. Kimingi, Kennedy Mwai, Kioko Mwikali, Cheryl Andisi, B. Kim Lee Sim, Philip Bejon, Melissa C. Kapulu, Samson M. Kinyanjui, Thomas Lavstsen, Abdirahman I. Abdi, CHMI-SIKA Study Team

**Affiliations:** a Centre for Geographic Medicine Research (Coast), Kenya Medical Research Institute, Wellcome Trust Research Programme, Kilifi, Kenya; b Centre for translational Medicine & Parasitology, Department of Immunology and Microbiology, University of Copenhagen and Department of Infectious Diseases, Righospitalet, Copenhagen, Denmark; c Pwani University Bioscience Research Centre, Pwani University, Kilifi, Kenya; d Sanaria Inc, Rockville, MD, USA; e Centre for Tropical Medicine and Global Health, Nuffield Department of Medicine, University Oxford, Oxford, United Kingdom; f School of Business Studies, Strathmore University, Nairobi, Kenya

**Keywords:** PfEMP1, VSA, Malaria, CHMI, Antibodies

## Abstract

**Objectives:**

Acquisition of antibodies to *Plasmodium falciparum* variant surface antigens (VSA) expressed on infected red blood cells (iRBCs) is associated with naturally acquired immunity to malaria. We have previously shown that antibodies to VSA on iRBCs are associated with protection against parasite growth in the context of controlled human malaria infection (CHMI). This study explored whether antibodies to recombinant antigens derived from *Pf*EMP1 domains were independently associated with protection during CHMI in semi-immune Kenyan adults.

**Methods:**

We used a multiplex bead assay to measure levels of IgG antibody against a panel of 27 recombinant *Pf*EMP1 antigens derived from the *Pf*EMP1 repertoire of the 3D7 parasite clone. We measured IgG levels in plasma samples collected from the CHMI participants before inoculation with Sanaria® PfSPZ Challenge, on the day of diagnosis, and 35 days post-inoculation. Univariable and multivariable Cox regression analysis was used to evaluate the relationship between the levels of antibodies to the antigens and CHMI outcome. We also adjusted for previous data including antibodies to VSA on iRBCs, and we assessed the kinetics of antibody acquisition to the different *Pf*EMP1 recombinant antigens over time.

**Results:**

All study participants had detectable antibodies to multiple *Pf*EMP1 proteins before inoculation. All *Pf*EMP1 antigens were associated with protection against parasite growth to the threshold criteria for treatment in CHMI, albeit with substantial collinearity. However, individual *Pf*EMP1 antigens were not independently associated with protection following adjustment for breadth of reactivity to VSA on iRBCs and schizont extract. In addition, antibodies to *Pf*EMP1 antigens derived from group B *Pf*EMP1 were induced and sustained in the participants who could not control parasite growth.

**Conclusion:**

This study shows that the breadth of antibody response to VSA on iRBCs, and not to specific *Pf*EMP1 antigens, is predictive of protection against malaria in CHMI.

## Highlights

• Naturally acquired Antibodies to recombinant *Pf*EMP1 were linked to protection in Controlled Human Malaria Infection (CHMI).
• Antibodies to the *Pf*EMP1 domains were highly correlated with each other and with antibody breadth to *P. falciparum* VSAs
• Breadth of antibody responses to VSAs on iRBCs is an independent predictor of protection in CHMI.

## Introduction

Malaria remains a significant global public health challenge, particularly in sub-Saharan Africa.^1^ In 2022, the WHO reported an estimated 249 million malaria cases and 608,000 deaths.^2^ *Plasmodium falciparum* transmitted by the anopheles mosquitoes, causes the most severe forms of malaria with the highest burden of the disease observed in children under five years of age and pregnant women.^3^, ^4^ Significant progress has been made in controlling the disease using insecticide-treated bed nets and indoor residual spraying, however, emerging resistance to insecticides and anti-malarial drugs continues to pose a challenge in malaria control efforts.^5^, ^6^, ^7^ The recommendations for RTS,S and R21 malaria vaccines by the WHO in malaria-endemic regions represent progress in the fight against malaria. However, the partial protection offered by these vaccines^8^, ^9^ warrants further studies on parasite biology and the human immune system to guide the development of next-generation malaria vaccines.

Individuals residing in malaria-endemic regions acquire immunity to symptomatic malaria.^10^, ^11^ This immunity is associated with gradually accumulating antibodies acquired following continued exposure to *Plasmodium falciparum*. Naturally acquired antibodies target antigens expressed during the blood stage of the parasite’s life cycle, including merozoites and variant surface antigens (VSAs) expressed on the infected erythrocyte surface.^12^, ^13^, ^14^ VSAs including *Plasmodium falciparum* erythrocyte membrane protein 1 (*Pf*EMP1), repetitive interspersed family (RIFIN) proteins, sub-telomeric variable open reading frame (STEVOR) proteins and surface-associated interspersed gene family (SURFIN) proteins are targets of naturally acquired immune response.^15^, ^16^, ^17^, ^18^

Of the variant antigens, *Pf*EMP1 is the most immunogenic surface antigen^19^ and is associated with immunity to malaria.^14^ *Pf*EMP1s are encoded by the *var* multi-gene family. Each parasite genome has 45–90 copies of *var* genes, whose expression occurs in a mutually exclusive manner.^20^ The extracellular portion of *Pf*EMP1 contains a combination of Duffy binding-like domain (DBL α, β, γ, δ, ε, and ζ) and cysteine-rich inter-domain regions (CIDR α, β, γ, δ).^21^, ^22^ These extracellular domains bind select human endothelial receptors to mediate sequestration of the parasite to the microvasculature, as an immune evasion mechanism allowing the parasite to escape splenic destruction. The *var* genes are classified as A, B, and C based on their upstream sequence, genomic location, and receptor binding phenotype of the encoded *Pf*EMP1.^23^, ^24^ Expression of group A and the chimeric group B/A *Pf*EMP1 that bind to human endothelial protein C receptor (EPCR) through CIDRα1 domain is associated with severe malaria.^25^, ^26^, ^27^, ^28^, ^29^ Conversely, expression of group B and C *Pf*EMP1 binding to CD36 through their CIDRα2–6 domains may be linked to uncomplicated malaria.^30^, ^31^ As *Pf*EMP1s binding to EPCR promotes endothelial inflammation, parasite sequestration via EPCR and ICAM1-binding group A and B/A *Pf*EMP1 have been hypothesized to support parasite growth and drive pathogenic inflammation in non-immune individuals.^32^

Despite the antigenic diversity that pose a challenge in targeting *Pf*EMP1 in vaccine development, evidence from several studies show that the clonal variation and polymorphisms of *Pf*EMP1 leads to the induction and accumulation of both cross-reactive and variant-specific antibodies with repeated *Plasmodium falciparum* infection.^33^, ^34^, ^35^, ^36^, ^37^ These naturally acquired antibodies have been shown to significantly reduce the risk of acquiring clinical malaria in children.^38^, ^39^

In previous work, we found that the breadth of antibody reactivity to variant surface antigens on infected red blood cells (iRBCs) using a panel of six different laboratory adapted parasite clones and *ex vivo* isolates was associated with protection in a controlled human malaria infection (CHMI) study including malaria-exposed participants.^40^ Here, using samples from the same CHMI study we measured antibody responses to a broad panel of recombinant proteins derived from various domains of 3D7 *Pf*EMP1s to investigate if specific *Pf*EMP1 domains could be more specifically linked to protection.

## Materials and methods

### Study design and population

This study was part of a larger controlled human malaria infection (CHMI) study described previously.^41^, ^42^ Briefly, 161 healthy adults with varying exposure and immunity to malaria were recruited in years 2016, 2017 and 2018 into three successive cohorts. The participants were drawn from areas with different malaria transmission intensities including Ahero in Western Kenya (moderate to high transmission), Kilifi South and Kilifi North at the Kenyan Coast, which have moderate and low transmission, respectively. The participants were injected by direct venous inoculation with 3.2 × 10^3^ Sanaria® PfSPZ Challenge (NF54).^43^, ^44^ After inoculation, parasite growth was monitored by qPCR twice daily from day 7 to day 14, and then once daily from day 15 to the end of the study on day 21. Participants who reached a parasitemia threshold of 500 *P. falciparum* parasites/μl were treated with anti-malarial drugs. Participants who developed malaria signs and symptoms at any stage of the study irrespective of their parasite density were also given anti-malarial drugs. All participants were treated 21 days post-inoculation irrespective of the study outcome.

### Multiplex immunoassays

**Scheme 1:**
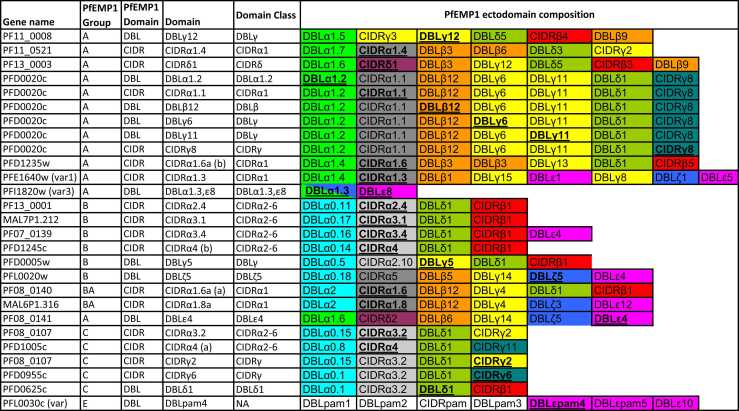
Recombinant *Pf*EMP1 domains were used as antigens in this study. The test domain position in *Pf*EMP1 and the *Pf*EMP1 sequence origin is given. Test antigens are marked bold and underlined.

A multiplex bead-based antibody assay was performed to measure IgG responses against recombinant *Pf*EMP1 protein domains **(**Scheme 1) derived from *Pf*EMP1 sequences of the 3D7 parasite strain, as previously described.^45^, ^46^, ^47^ The plasma samples used for this study were collected from participants before the challenge (C-1), at the day of diagnosis which was the day individual participants reached a threshold for treatment, and 35 days post-inoculation (C+35). Briefly, the plasma samples were assayed once each, diluted 1:80 in assay buffer E (ABE: 0.1% BSA, 0.05% Tween-20 in PBS, pH 7.4). 50 μl beads and 50 μl diluted plasma were added to 96-well microtiter plates (MSBVS 1210, MilliporeSigma). 50 μl of PE-conjugated Goat Anti-Human IgG (AB_2337681; Jackson ImmunoResearch Laboratories) diluted at 1:3000 was added. Median fluorescent intensities were measured using the Luminex 200 system, with reader set to read a minimum of 100 beads per bead region. Pooled malaria hyperimmune plasma was serially diluted in ABE at 1:50, 1:158, 1:500, 1:1580, 1:5000, 1:15,800, 1:50,000, and 1:158,000 and were included on every plate as standards. The standard curve for each antigen was used to interpolate IgG concentrations from the test plasmas, reported as arbitrary units.

### Detection of antibodies to *P. falciparum* variant surface antigens and schizont extract

The levels of antibodies to the VSAs on iRBCs present in the plasma of CHMI study participants a day prior to the challenge (C-1) were quantified via flow cytometry as previously described.^40^ Briefly, six *P. falciparum* isolates, two long-term laboratory-adapted isolates and four heterologous clinical isolates obtained from acute infections but matured ex-vivo to the trophozoite stages were used. RBCs infected with the trophozoite stages of the isolates were incubated with the plasma samples, and antibody reactivity to iRBCs was detected using fluorescein isothiocyanate (FITC) conjugated sheep anti-human IgG. Antibodies to the *P. falciparum* schizont extract were detected using ELISA as previously described.^48^ Briefly, *P. falciparum* 3D7 parasites were grown to schizont stage, followed by a series of freeze-thaw cycles to obtain the schizont extract. Antibodies to the schizont extract were detected using the standard ELISA protocol.

### Statistical analysis

Data analysis and visualization were performed in R Statistical Software (version 4.3.1; R Core Team 2023) using the *tidyverse*. *Survival* and *glmnet* packages were used for Cox regression analysis. Statistical significance was defined as a P value <0.5, as indicated in the figure legends.

To identify the *Pf*EMP1 domains that were independently associated with protection from the risk of reaching the study-determined threshold for treatment, we performed a regularized Cox regression analysis with the least absolute shrinkage and selection operator (LASSO) penalty using 10-fold cross-validation with 1000 iterations.^49^

To evaluate the relationship between *Pf*EMP1 antibody breadth and CHMI clinical outcome, we developed an antibody response score for the 27 antigens. Each participant’s antibody response to a particular antigen was scored as either 0,1or 2 depending on whether the response was in the lower, middle, or upper tertile of the responses to that antigen. The total score across the 27 proteins (minimum=0, maximum=54) was taken as the individual’s *Pf*EMP1 antibody breadth and was further classified as low breadth (total score=<27) or high breadth (total score >27).

## Results

### CHMI outcome

161 participants were recruited into the study. However, as reported previously,^42^ 19 participants were excluded from downstream analysis either because of detection of anti-malaria drugs in plasma samples collected prior to the challenge, or because of detection of other parasite strains other than the NF54 that was used to inoculate all the participants. As done previously,^40^, ^50^, ^51^ we therefore conducted analysis on 142 participants, who were grouped as either untreated PCR negative (n = 33), untreated PCR positive (n = 53), treated non-febrile (n = 30), or treated febrile (n = 26).^42^, ^48^

### CHMI study participants exhibited high and broad antibody reactivity to *Pf*EMP1 antigens prior to infection

**Fig. 1:**
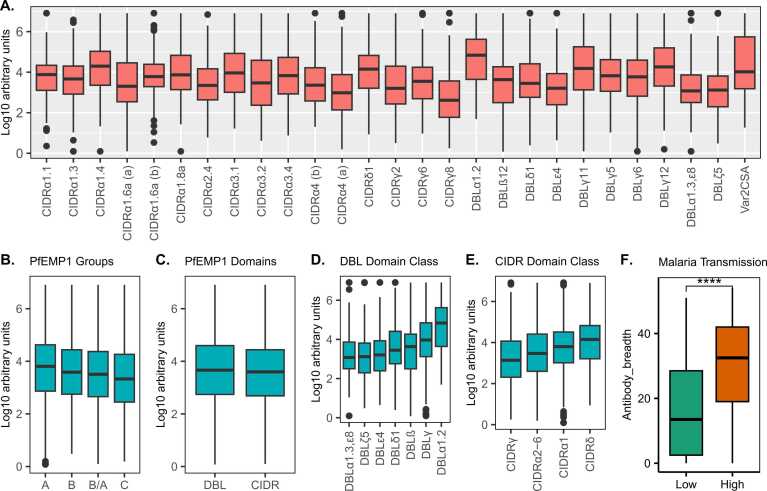
Magnitude and breadth of *Pf*EMP1 domain IgG antibodies before CHMI. Antibody reactivity to individual recombinant *Pf*EMP1 domains (A), *Pf*EMP1 Groups (B), *Pf*EMP1 DBL and CIDR Domains (C) and DBL and CIDR domain classes (D &E) and antibody breadth stratified by malaria transmission intensity (F). ****P-value = <0.0001.

To determine the *Pf*EMP1 IgG antibody profile of the study participants before the challenge, we used a Luminex bead-based assay to measure antibody reactivity to 27 recombinant *Pf*EMP1 CIDR and DBL protein domains sampled across the *Pf*EMP1 repertoire of the NF54/3D7 parasites (Scheme 1). Six recombinant proteins were derived from the PFD0020c *Pf*EMP1, whose expression showed a significant association with parasite multiplication rate in a previous CHMI study.^52^ All samples passed the quality control criteria. The study participants showed varying recognition of the *Pf*EMP1 proteins, but with no *Pf*EMP1 domains recognized at higher levels than others (Fig. 1A-E). As the participants were drawn from malaria endemic areas with different malaria transmission intensities, we stratified the antibody response to *Pf*EMP1 proteins by residence at high vs. low transmission intensity. In line with previous immuno-epidemiologic studies,^53^ individuals from high malaria transmission areas had higher levels of IgG antibodies to all *Pf*EMP1 domains tested compared to individuals from low malaria transmission regions (Supplementary Figure 1). Additionally, the overall breadth of IgG reactivity to the *Pf*EMP1 domains was significantly higher in participants residing in high malaria transmission regions, compared to those from low malaria transmission regions (Fig. 1F).

### Naturally acquired *Pf*EMP1 antibodies are associated with protection against reaching treatment criteria in CHMI

**Fig. 2:**
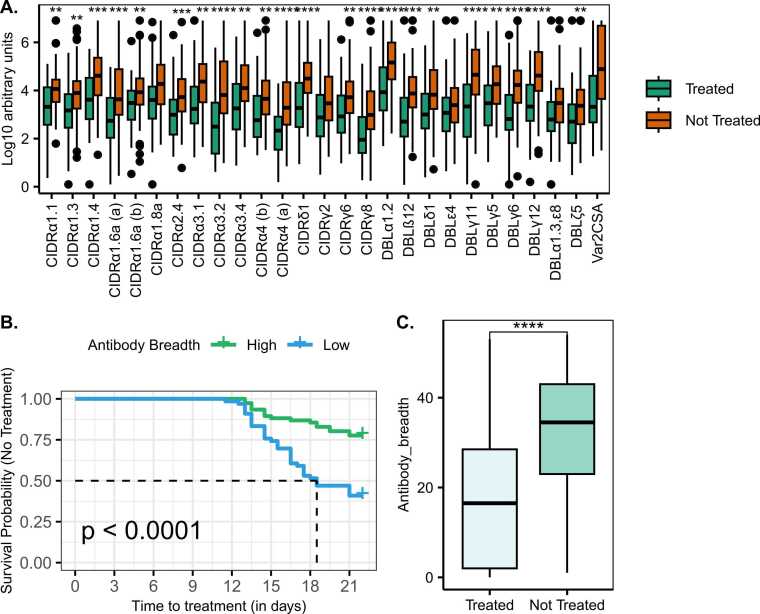
*Pf*EMP1 antibody magnitude and breadth were associated with protection against the risk of reaching the threshold for treatment after infection. **A.** Comparison of pre-existing *Pf*EMP1 antibody levels between the treated and untreated groups. **B.** Kaplan Meier survival analysis of time to treatment stratified by breadth of antibodies to *Pf*EMP1-derived recombinant antigens before infection. The dotted line indicates the median survival time where the survival probability is below 50%. *Pf*EMP1 antibody breadth stratified by CHMI outcome. **C.** CHMI clinical outcome stratified by antibody breadth. *P-value < 0.05, **P-value < 0.01, ***P-value < 0.001, ****P-value = <0.0001.

To investigate the association between naturally acquired *Pf*EMP1 IgG antibodies and the clinical outcome following the sporozoite challenge, we performed univariable Cox regression analyses to determine the hazard ratios associated with the antibody response as the independent variable and the time to treatment as the dependent variable. All the *Pf*EMP1 domains tested were significantly associated with protection against parasite growth reaching the threshold for treatment following CHMI (Supplementary Table 1, Fig. 2A), and the breadth of *Pf*EMP1 antibodies prior to infection was also significantly associated with protection (Fig. 2B and C).

### Multivariable analysis of naturally acquired antibodies

As substantial collinearity was detected in the univariable analysis (Supplementary Figure 2), we then sought to identify antibody responses independently predicting protection against reaching the threshold for treatment. *Pf*EMP1 responses were subjected to a least absolute shrinkage and selection operator (LASSO) penalized regression analysis using an alpha value of one and minimum lambda. The regularized Cox regression was used to select the most important predictors to avoid overfitting.^49^ Antibodies to four *Pf*EMP1 domains CIDRα3.2, CIDRα4(a), DBLγ11 and DBLγ12 were selected as top predictors (Supplementary Figure 3). The four selected variables were then included in a multivariable model in which only the CIDRα3.2 showed an independent association with protection (Table 1). We then included antibody responses to CIDRα3.2, CIDRα4(a), breadth of antibody to recombinant PfEMP1 domains, VSA on iRBCs and schizont extract that we previously reported to be associated with protection^40^ in a multivariable model. Only the breadth of antibody reactivity to VSA on iRBCs and anti-schizont showed significant independent association with protection against parasite growth (Table 1). To validate the variables selected by LASSO using alternate statistical approach, we performed a multivariable analysis by fitting responses to antigens in *Pf*EMP1 group A, B, B/A and C in separate models (Supplementary Table 2). The top predictors selected from the *Pf*EMP1 groups models were CIDRα1.6(a), CIDRα3.2, CIDRα4(a) and DBLγ12. We included the selected top predictors in a multivariable model together with breadth of antibody reactivity to *Pf*EMP1-derived recombinant proteins, VSA on iRBCs and schizont extract. Similar to our results above, only breadth of antibody reactivity to VSA on iRBCs and schizont extract showed independent protection against reaching the threshold for treatment with anti-malaria drugs (Supplementary Table 3).

**Table 1:** Cox regression analysis of the association between antibody response to the variables selected by LASSO and the time to reaching the study determined threshold for treatment.

	Selected by LASSO			Multivariable		Including PfEMP1 and iRBC breadth and Schizont Extract		
Variable	HR (95%CI)	P value	Variable	HR (95%CI)	P value	Variable	HR (95%CI)	P value
CIDRα3.2	0.63 (0.4−0.98)	0.04	CIDRα3.2	0.54 (0.36−0.82)	0.003	CIDRα3.2	0.82 (0.49−1.38)	0.46
CIDRα4(a)	0.79 (0.51−1.22)	0.29	CIDRα4(a)	0.66 (0.45−0.97)	0.03	CIDRα4(a)	0.74 (0.44−1.25)	0.26
DBLγ11	0.83 (0.53−1.32)	0.43				PfEMP1 antibody breadth	1.21 (0.68−2.12)	0.52
DBLγ12	0.85 (0.52−1.39)	0.52				VSA on iRBC antibody breadth	0.5 (0.32−0.77)	0.002
						Schizont	0.45 (0.27−0.76)	0.003

### *Pf*EMP1 IgG antibodies are rapidly induced following infection with *P. falciparum* sporozoites

**Fig. 3:**
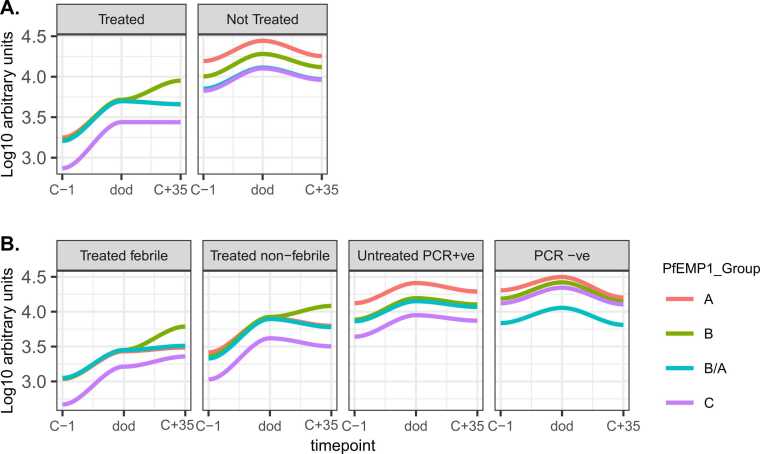
*Pf*EMP1 antibody kinetics following infection with *P. falciparum* sporozoites. A and B: antibody reactivity to *Pf*EMP1 antibodies at C-1, dod, and C+ 35 with LOESS fit curves stratified by CHMI study outcomes.

To investigate *Pf*EMP1 antibody kinetics, we analyzed the antibody magnitude before infection (C-1), at the day of diagnosis (dod), which is a varying time point of drug treatment irrespective of the study outcome and at the end of the study, and 35 days post-infection. Antibodies to the *Pf*EMP1 antigens generally increased shortly after infection (at dod) across all *Pf*EMP1 domains and *Pf*EMP1 groups (Fig. 3A and B, Supplementary Figure 4). Interestingly, antibodies to a few *Pf*EMP1 group B derived antigens continued to be induced in the treated group (Fig. 3A and B), while in the untreated group, the antibody levels to all *Pf*EMP1 antigens started to wane after the day of diagnosis.

## Discussion

Harnessing a controlled human malaria infection study on healthy Kenyan adults with varying degrees of naturally acquired immunity to malaria, the aim of the study was to interrogate the relationship between naturally acquired antibodies to specific antigens derived from 3D7 *Pf*EMP1 proteins and the clinical outcomes after infection, and to longitudinally analyze specific *Pf*EMP1 antibody kinetics post-infection.

We observed that individuals who were protected in CHMI (i.e. did not reach a study-determined threshold for treatment) had statistically significant higher levels of *Pf*EMP1 antibodies before commencing CHMI. There was substantial collinearity among the various antibody responses, and we found that protection against parasite growth during the CHMI study was independently associated with the breadth of antibodies against *P. falciparum Pf*EMP1 antigens. There were two individual *Pf*EMP1 domains that stood out as having independent associations with protection against other *Pf*EMP1 domains (i.e. CIDRα3.2, CIDRα4(a), as shown in Table 1, middle columns). However, these individual *Pf*EMP1 domains did not show independent associations following adjustment for antibody breadth and schizont extract. A similar conclusion was drawn from our previous analysis of the CHMI participant’s antibody responses to a panel of parasite-infected erythrocytes.^40^ Moreover, when we included antibodies to VSA on iRBCs from this previous study, breadth of response to VSA on iRBCs and response to schizont extract were the independent correlates of protection over responses to specific *Pf*EMP1 domains, indicating that CHMI protection is associated with broad immune response to *P. falciparum* iRBC surface antigens in general. Several possibilities could explain our finding that antibody breadth to VSA on iRBCs is independently associated with protection rather than individual *Pf*EMP1 domains. The antibody recognition of the VSA on iRBCs may be measuring a broader diversity of variant surface antigens expressed on the iRBC than those included among the *Pf*EMP1 domains. It is also possible that the breadth of antibody recognition to VSA on iRBCs is an indication of antibody response to other yet-to-be-identified antigen(s) or less studied VSA on iRBCs that elicit strong protective immunity against reaching the treatment threshold in CHMI. Finally, it is possible that antibodies to VSA on iRBCs identify conformation-dependent binding, which is not captured in recombinant proteins, although the *Pf*EMP1 domains were produced recombinantly in insect cells, a method previously shown to ensure high success rates in functional, folded proteins.^54^, ^55^

Longitudinal analysis of *Pf*EMP1 antibody kinetics showed induction of *Pf*EMP1 antibodies to all antigens as well as to *Pf*EMP1 groups post-infection. However, the kinetics of acquisition differed across *Pf*EMP1 groups and across the study clinical outcome categories. In the treated group, induction of antibodies to *Pf*EMP1 Group B was robust and sustained over time compared to the untreated group. This is an interesting finding which suggests that the infecting strain, NF54, expressed group B *var* genes post-infection in the treated group perhaps because of a gap in the repertoire of antibodies to group B *Pf*EMP1.

A limitation of this study is that we did not quantify the *var* transcripts from participants who had PCR-detectable parasitemia. This would have enabled us to corroborate our findings from the serology data in the susceptible group. However, previous CHMI studies in malaria naïve and malaria exposed cohorts reported similar findings of dominant expression of group B *var* genes post infection.^56^, ^57^

Although most CHMI studies have been conducted using the NF54 or 3D7 clones of *P. falciparum*, recent research has begun to explore the use of other strains from different regions such as 7G8 from Brazil, NF166. C8 from Guinea, and NF135. C10 from Cambodia. One study by Bachmann et al. aimed to investigate if the expression of *var* genes differed depending on the infecting strain. To do this, they evaluated the expression of *var* genes following experimental infection of malaria-naïve volunteers with a PfSPZ Challenge (7G8) parasite clone.^58^ The study reported that at the onset of a 7G8 blood stage infection, there was a broad activation of all *var* genes, with a predominance of group B *var* genes.^59^ However, while this may represent a bet-hedging strategy at liver release, the observation that antibodies to group B *Pf*EMP1 domains were acquired only in the treated group, indeed reflects that a broad antibody coverage of the group B *Pf*EMP1 is slowly acquired or maintained through repeated exposure.

In conclusion, the evidence is that the breadth of response of VSA on iRBC is independently associated with protection in a CHMI study, and that antibody responses to specific *Pf*EMP1 domains are associated with protection only through cross-correlations, and hence did not show independent association.

## Funding

This work was funded by the 10.13039/100004440Wellcome Trust (107499 and 209289). The funder had no role in the design, analysis, write up, or decision to submit for publication.

## Ethics statement

The study was conducted at the Kenya Medical Research Institute-Wellcome Trust Research Programme in Kilifi, Kenya, and Ethical approval was obtained from KEMRI Scientific and Ethics Review Unit (KEMRI//SERU/CGMR-C/029/3190) and the University of Oxford Tropical Research Ethics Committee (OxTREC 2–16). The clinical trial where samples were analyzed was registered on ClinicalTrials.gov (NCT02739763).

## Author contribution

AA, TL, CK, MK and PB designed the study. AK, HK, KM, KM, and AA analyzed the data. AK and AA wrote the first draft of the manuscript. PB, MK, SK, AA, and CK edited the manuscript. LT and TL performed Luminex assays. All authors contributed to the interpretation of the analyses and revised the draft manuscript.

## Declaration of Competing Interest

The authors declare the following financial interests/personal relationships which may be considered as potential competing interests: B Kim Lee Sim and members of the CHMI-SIKA Study Team (Yonas Abebe, Stephen L. Hoffman, Eric R. James, Thomas L. Richie, and Peter F. Billingsley are salaried, full-time employees of Sanaria, the manufacturer of Sanaria PfSPZ Challenge (NF54).

## Data Availability

Data will be deposited into the KWTRP Harvard Dataverse repository. The data will be available to researchers who will need to submit proposals to gain access to the data following a signed data access agreement.

## Appendix A

**Members of the CHMI-SIKA Study Team**

Centre for Geographic Medicine Research (Coast), Kenya Medical Research Institute-Wellcome Trust Research Programme, Kilifi, Kenya: Abdirahman I Abdi, Philip Bejon, Zaydah de Laurent, Mainga Hamaluba, Domtila Kimani, Rinter Kimathi, Kevin Marsh, Sam Kinyanjui, Khadija Said Mohammed, Moses Mosobo, Janet Musembi, Jennifer Musyoki, Michelle Muthui, Jedidah Mwacharo, Kennedy Mwai, Joyce M Ngoi, Omar Ngoto, Patricia Njuguna, Irene Nkumama, Francis Ndungu, Dennis Odera, Donwilliams Omuoyo, Faith Osier, Edward Otieno, Jimmy Shangala, James Tuju, Juliana Wambua, Thomas N Williams.

Sanaria Inc, Rockville, MD, United States: Yonas Abebe, Peter F Billingsley, Stephen L Hoffman, Eric R James, Thomas L Richie, B Kim Lee Sim.

Centre for Tropical Medicine and Global Health, Nuffield Department of Medicine, University Oxford, Oxford, United Kingdom: Sam Kinyanjui, Kevin Marsh.

Department of Pathology, University of Cambridge, Cambridge, United Kingdom: Peter C Bull.

Pwani University, P. O. Box 195–80108, Kilifi, Kenya: Sam Kinyanjui, Cheryl Kivisi.

Epidemiology and Biostatistics Division, School of Public Health, University of the Witwatersrand, Johannesburg, South Africa: Kennedy Mwai.

Centre for Infectious Diseases, Heidelberg University Hospital, Heidelberg, Germany: Irene Nkumama, Dennis Odera, Faith Osier.

Center for Research in Therapeutic Sciences, Strathmore University, Nairobi, Kenya: Bernhards Ogutu, Fredrick Olewe, John Ong’echa.

Center for Research in Therapeutic Sciences, Strathmore University, Nairobi, Kenya: Bernhards Ogutu, Fredrick Olewe, John Ong’echa.
