# Supplementary material for: Antibodies to PfEMP1 and variant surface antigens: Protection after controlled human malaria infection in semi-immune Kenyan adults
Source: J Infect. Author manuscript; Available in PMC 2024 Oct 1. (PMC11409615; doi:10.1016/j.jinf.2024.106252)
Supplement: Supplementary Material [file EMS198792-supplement-Supplementary_Material.docx]

**SUPPLEMENTARY MATERIAL**


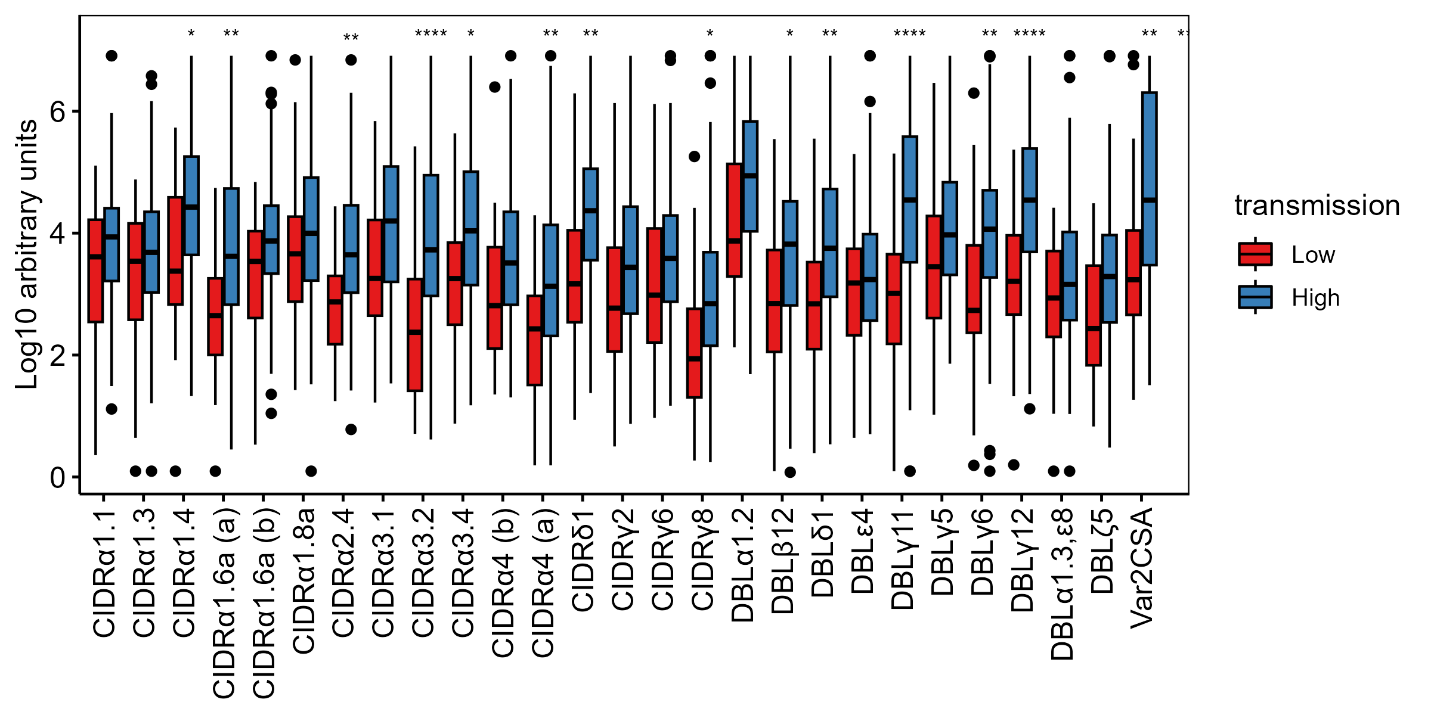


**Supplementary Figure 1:** IgG antibody responses to *Pf*EMP1 antigens at C-1 stratified by malaria transmission intensity. * P-value <0.05, ** P-value <0.01, *** P-value <0.001 **** - P-value = <0.0001

**Supplementary Table 1:** Univariate Cox regression analysis on *Pf*EMP1 IgG antibodies and the risk of reaching a threshold for treatment.


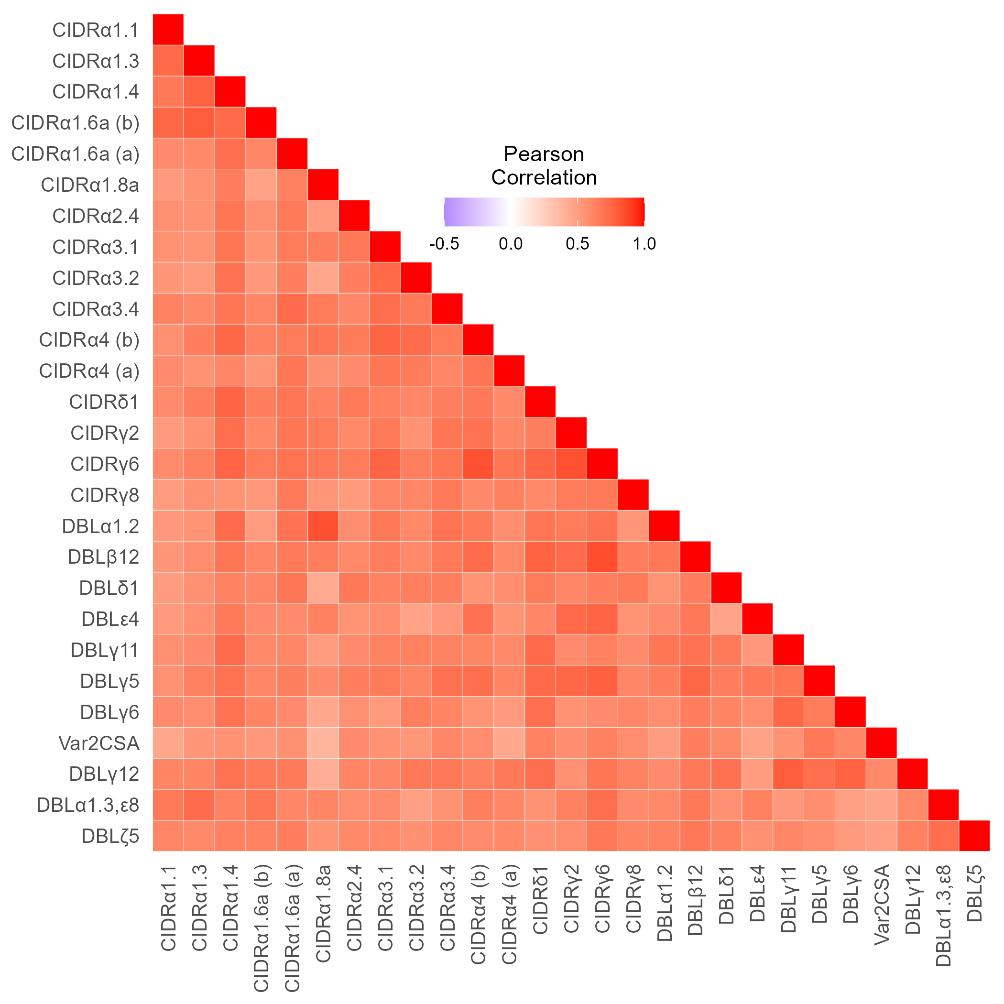


**Supplementary Figure 2:** Correlation matrix of antibody responses to *Pf*EMP1 antigens prior to the challenge.


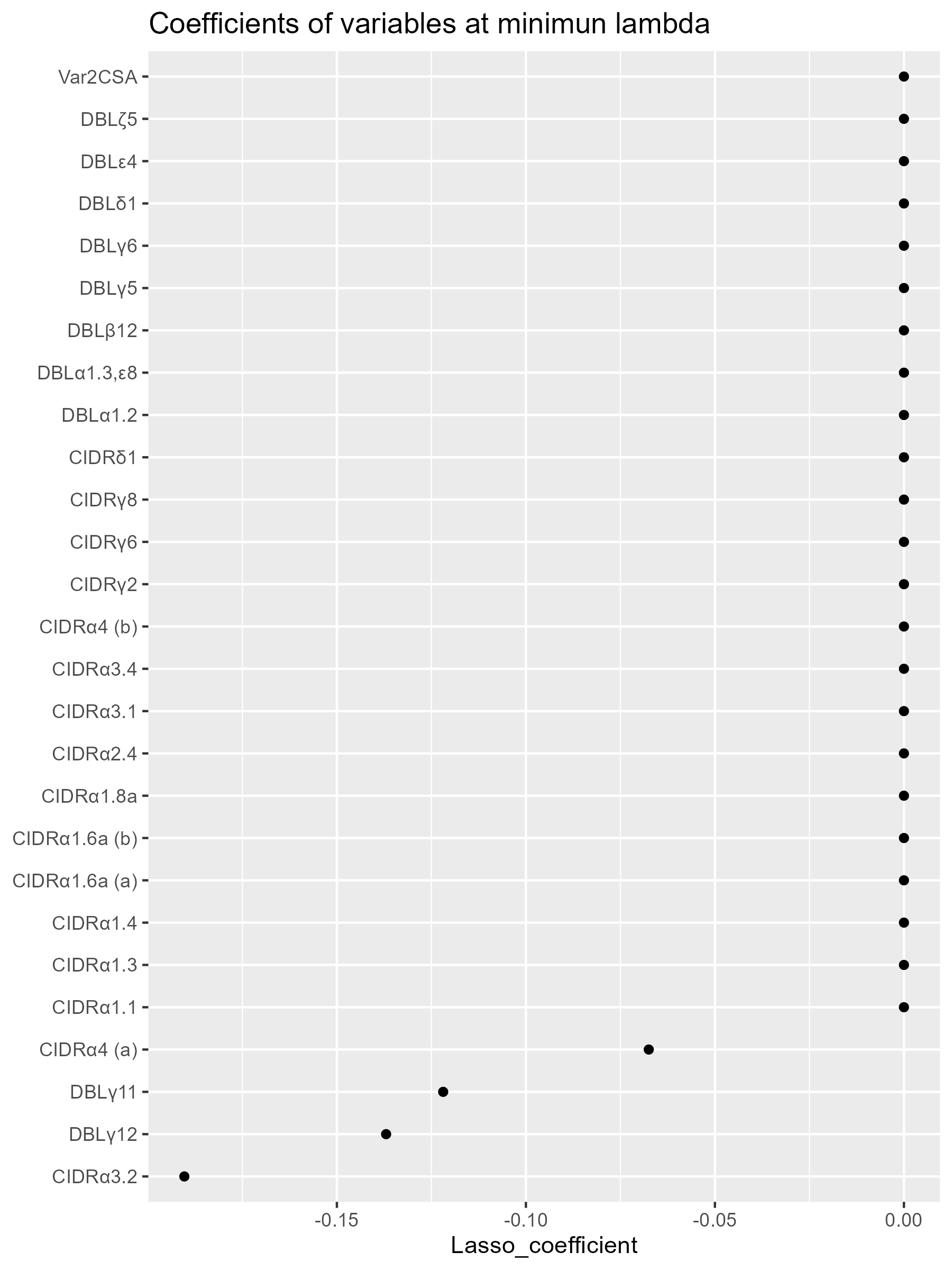


**Supplementary Figure 3:** Feature selection using Lasso penalized regression.

**Supplementary Table 2:** Multivariable Cox regression analysis on *Pf*EMP1 IgG antibodies stratified by PfEMP1 groups and the risk of reaching a threshold for treatment.

**Supplementary Table 3:** Multivariable Cox regression analysis including antibody breadth to *Pf*EMP1, VSA on iRBC, Schizont extract and the risk of reaching a threshold for treatment.


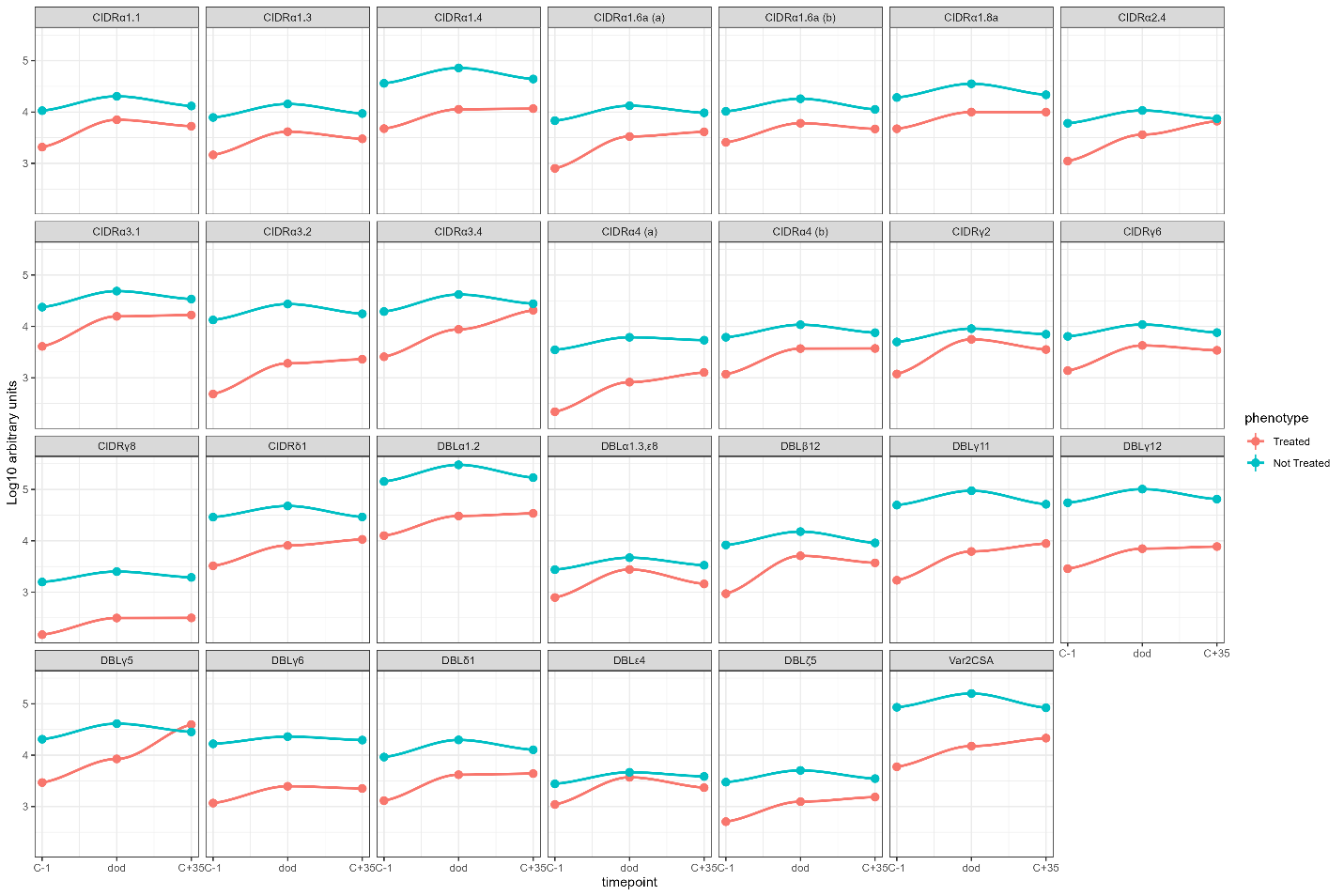


**Supplementary Figure 4:** *Pf*EMP1 antibody kinetics: Longitudinal analysis of *Pf*EMP1 specific antibodies before the challenge, at the day of diagnosis and 35 days post infection. LOESS fit curves stratified by treatment outcome.
